# Eating at food outlets and leisure places and “on the go” is associated with less-healthy food choices than eating at home and in school in children: cross-sectional data from the UK National Diet and Nutrition Survey Rolling Program (2008–2014)

**DOI:** 10.1093/ajcn/nqy057

**Published:** 2018-05-07

**Authors:** Nida Ziauddeen, Polly Page, Tarra L Penney, Sonja Nicholson, Sara FL Kirk, Eva Almiron-Roig

**Affiliations:** 1 Medical Research Council (MRC) Elsie Widdowson Laboratory, Cambridge, United Kingdom; 2 Academic Unit of Primary Care and Population Sciences, Faculty of Medicine, University of Southampton, Southampton, United Kingdom; 3 UKCRC Center for Diet and Activity Research (CEDAR), MRC Epidemiology Unit, University of Cambridge School of Clinical Medicine, Institute of Metabolic Science, Cambridge, United Kingdom; 4 School of Health and Human Performance, Dalhousie University, Halifax, Nova Scotia, Canada; 5 Center for Nutrition Research, University of Navarra, Pamplona, Spain

**Keywords:** eating location, core food, noncore food, adolescents, home meals, school food

## Abstract

**Background:**

Where children eat has been linked to variations in diet quality, including the consumption of low-nutrient, energy-dense food, a recognized risk factor for obesity.

**Objective:**

The aim of this study was to provide a comprehensive analysis of consumption patterns and nutritional intake by eating location in British children with the use of a nationally representative survey.

**Design:**

Cross-sectional data from 4636 children (80,075 eating occasions) aged 1.5–18 y from the UK National Diet and Nutrition Survey Rolling Program (2008–2014) were analyzed. Eating locations were categorized as home, school, work, leisure places, food outlets, and “on the go.” Foods were classified into core (considered important or acceptable within a healthy diet) and noncore (all other foods). Other variables included the percentage of meals eaten at home, sex, ethnicity, body mass index, income, frequency of eating out, takeaway meal consumption, alcohol consumption, and smoking.

**Results:**

The main eating location across all age groups was at home (69–79% of eating occasions), with the highest energy intakes. One-third of children from the least-affluent families consumed ≤25% of meals at home. Eating more at home was associated with less sugar and takeaway food consumption. Eating occasions in leisure places, food outlets, and “on the go” combined increased with age, from 5% (1.5–3 y) to 7% (11–18 y), with higher energy intakes from noncore foods in these locations. The school environment was associated with higher intakes of core foods and reduced intakes of noncore foods in children aged 4–10 y who ate school-sourced foods.

**Conclusions:**

Home and school eating are associated with better food choices, whereas other locations are associated with poor food choices. Effective, sustained initiatives targeted at behaviors and improving access to healthy foods in leisure centers and food outlets, including food sold to eat “on the go,” may improve food choices. Home remains an important target for intervention through family and nutrition education, outreach, and social marketing campaigns. This trial was registered with the ISRTCN registry (https://www.isrctn.com) as ISRCTN17261407.

## INTRODUCTION

Poor diet in childhood and adolescence has been recognized as a risk factor for obesity and associated conditions during adulthood (1, 2). The food environment is an important determinant of children's dietary behavior (3–5), and therefore improvements in food environments could facilitate healthier eating behaviors (2, 6). Specifically, eating out-of-home in children has been linked to the consumption of nutrient-poor, energy-dense foods (7, 8), also known as “noncore foods,” including sugar-sweetened beverages (SSBs), cakes, and potato chips.

Research into the home environment has shown higher intakes of desirable nutrients such as fiber and lower intakes of noncore foods when eating at home than at other locations, paired with lower dietary energy density and percentage of energy from fat (8, 9). However, home may also represent a setting for the consumption of less-healthy food, including pizza and energy-containing beverages bought at stores (10), food from takeaway restaurants (11), and snacks (12), particularly in young people (8). In Europe, eating at home is still associated with higher intakes of desirable nutrients and lower dietary energy density in children and adolescents (8, 13).

The school food environment is also important because children spend a considerable part of the day in structured education where they are exposed to many influential factors, such as food cues, peer pressure, and after-school activities (13, 14). The introduction of school food policies and meal standards may have played a positive role (15, 16), but progress is slow (17, 18) and school meals are not consistently associated with improved intakes across countries (13, 18).

The consumption of food away from home or school has particularly been in the spotlight (8–11, 19–22). Previous research in children in this field has been focused in the United States using restaurant menu offerings or food-purchasing data (10) and consumption in specific locations only (4, 19, 21) or from specific food groups (7), and in the United Kingdom on the frequency of consumption in home locations only (11) or for specific food or age groups only (8, 14).

The objective of the present study was to carry out an in-depth analysis of the food environment of children and adolescents in the United Kingdom, with a particular focus on the location of eating and the types of food consumed in each location, with the use of cross-sectional dietary data across a wide range of locations and to investigate potential modulatory factors. Our research questions were as follows: *1*) what are the most common eating locations for children, *2*) what types of foods are consumed in each location and how much do they contribute to daily energy intakes, and *3*) how do these patterns fluctuate with modulatory factors, in particular whether income, individual traits, and drinking and smoking habits are significant predictors of eating location patterns.

## METHODS

### Sample

We conducted a secondary analysis on data collected between 2008 and 2014 as part of the National Diet and Nutrition Survey Rolling Program (NDNS RP; years 1–6) (23). The NDNS RP was designed to assess the diet, nutrient intake, and nutritional status of the general population aged ≥1.5 y living in private households in the United Kingdom and includes a nationally representative sample. The survey aims to recruit a core sample of 1000 participants/y (500 children aged 1.5–18 y and 500 adults) and a boost sample of ≤600 participants/y. Details on the survey design and sampling methods of the NDNS RP have been published elsewhere (24). Briefly, a random sample was drawn from the Postcode Address File, a list of all addresses in the United Kingdom. Addresses were clustered into primary sampling units, smaller geographical areas based on postcode sectors, which were randomly selected from across the United Kingdom. From each primary sampling unit, 27 addresses were randomly selected, and information describing the purpose of the study was posted to the selected addresses. Interviewers then contacted these addresses to recruit participants and place diet diaries. An average response rate of 54% was achieved, which is just under the expected response rate of 55% on the basis of response rates to other similar surveys (25). This trial was registered with the ISRTCN registry as ISRCTN17261407 (https://www.isrctn.com).

### Dietary data

Dietary assessment was carried out by using 4-d estimated diaries; participants were asked to keep a record of everything they ate and drank over 4 consecutive days. Participants were assigned random start days for the diary and thus could include weekdays only or week- and weekend days. All of the participants who completed 3 or 4 diary days were included (2%; 84 participants completed 3 diary days). For children aged ≤12 y, the parent or caregiver was asked to complete the diary with input from the child as appropriate. Participants were asked to record all foods and beverages consumed, including brand names, recipes for home-cooked foods, and information on portion sizes. Portion sizes were generally estimated and recorded in household measures (spoonfuls, glasses, cups) and were informed with the use of standardized pictures provided in the front of the diary, food labeling information, or proportions of recipes when provided. Participants were also asked to return the packaging of any branded items consumed with the diary to enable use of the weights and nutrition information on the label by the researchers during data coding.

Trained interviewers undertook 3 visits with each participant. At the first visit, the interviewer administered the Computer-Assisted Personal Interview (CAPI) and provided the diary. This was followed by a brief visit to provide support during completion and to check for compliance. At the third visit, the interviewer reviewed and edited the diary for possible omissions together with the respondent and collected the diary.

Diaries were coded by trained coders and processed using DINO (Diet In Nutrients Out) (26). Each recorded item was assigned a suitable food and portion code. The food-composition data used were from the Department of Health's NDNS Nutrient Databank, and portion sizes were from the Food Standard Agency's portion-size book (27), plus published age-appropriate portion sizes for children (28) when standard portion sizes were recorded in the diary using the pictures provided. For composite items that could be divided into their component parts (e.g., sandwiches), each individual component was coded separately. This approach was also applied to homemade dishes for which recipes had been provided in the diary, and these were then linked together to indicate being cooked together.

For validation of estimations of energy intake from the self-reported dietary records of food and beverages consumed, the NDNS RP included a doubly labeled water (DLW) substudy in participants aged ≥4 y (24). The results of the DLW subsample analysis indicated that reported energy intake is 12% below total energy expenditure in children aged 4–10 y and 26% lower in children aged 11–15 y. Factors that may contribute to this difference include misreporting of actual consumption, the possibility that participants underreported or changed their usual intake during the diary period (2–3 wk before DLW measurement), and methodologic considerations related to dietary assessment method, food composition, and portion-size assignment.

### Other variables

BMI was calculated by using height and weight measurements taken by the interviewer. Data on ethnicity, income, frequency of takeaway meal consumption, frequency of eating out, and frequency of drinking and smoking were collected through self-report with the use of questions that were designed specifically for the NDNS CAPI. Due to the small number of participants in the mixed, black, Asian, and other ethnic groups, this was reported as a combined group of nonwhites. The questions used were as follows: “On average, how often do you/does child eat take-away meals at home?” and “On average, how often do you/does child eat meals out in a restaurant or cafe?” In both questions, the interviewer specified that “‘meals’ means more than a beverage or bag of chips” and participants were asked to “include pizza, fish, and chips, Indian, Chinese, burgers, kebab, etc.” Response options available to the participant were “rarely or never,” “1–2 times per month,” “1–2 times per week,” “3–4 times per week,” and “5 or more times per week.”

In addition, participants aged 8–24 y were given the option of filling out a self-completion booklet or answering the interviewer’s CAPI questions for 11 smoking and 42 alcohol consumption questions to determine units, nature, and frequency of drinking. Information on smoking and drinking behavior was provided by 97% of children aged 8–18 y.

### Defining eating occasions

In addition to details on what and how much food or beverage was consumed, for each diary entry NDNS RP participants were asked to record the following: where they were, who they were eating with, and whether they were watching television or sitting at a table. Individual food data by time slot were analyzed. Eating occasions were defined as consecutive diary entries that were recorded within 15 min of each other for the same location (29). For the purpose of our study, detailed eating location was aggregated into 6 broad categories as follows:

- Home: bedroom, dining room, garden, kitchen, living room, home-other, home-unspecified (excludes other people's homes)
- School: all school cafeteria categories, classroom, school-other, playground, nursery or kindergarten
- Leisure places: sports clubs, sports leisure venue, leisure activity place, cinema, shopping center, place of interest, attractions, community or day center
- Food outlets: restaurant/pub/nightclub, fast-food outlets, coffee shop/cafe/deli/sandwich bar
- “On the go”: bus/car/train, outside-other, street
- Work: all work cafeteria categories, desk, work-other

Eating occasions at other locations (5.4% of all occasions) such as friends’ and relatives’ house, caregivers’ home, holiday accommodation, other place, and place of worship were excluded from this analysis because these represented a nonhomogeneous mixture of locations. Unspecified locations included home-unspecified (categorized as home) or unspecified (excluded 1.4% of all occasions). The number of entries in each of these locations was not sufficient to divide into separate analyses and therefore does not allow for meaningful interpretation.

### Dietary variables—core and noncore foods

Each food consumed in the NDNS RP was defined as a core or noncore food on the basis of a previously published categorization (30) (**Table 1**). In line with Johnson et al. (30), core foods were defined as those included in the principal food groups and considered important or acceptable within a healthy diet, such as cereals and cereal products, meat (excluding processed meat), meat alternatives, fish, vegetables, fruit, nuts and seeds, and dairy products (30, 31). All other foods were classified as nonessential (“noncore”) foods and included pastries, cakes, high-fat snacks, and sugary drinks among other foods (30).

**TABLE 1:** NDNS RP food groups defined as core and noncore^1^

Core foods	Noncore foods
Pasta, rice, and other miscellaneous cereals	Biscuits/cookies
Bread (all types)	Buns, cakes, pastries, and fruit pies
Breakfast cereals (all types)	Puddings
Milk (all types)	Ice cream
Cheese	Butter, spreads, and oil
Yogurt	Dairy desserts
Eggs and egg dishes	—
Beef, veal, and dishes	Meat pies and pastries
Lamb and dishes	—
Pork and dishes	Bacon and ham
Chicken and turkey dishes	Coated chicken and turkey
Liver products and dishes	—
—	Burgers and kebabs
—	Sausages
—	Other meat and meat products
White fish, shellfish, and fish dishes	Coated or fried white fish
Oily fish	—
Salad and other raw vegetables	—
Vegetables (not raw) including beans and meat alternatives	—
Other potatoes and potato salads	Chips, fried and roast potatoes, and potato products
Nuts and seeds	—
Fruit	—
Smoothies	—
Fruit juice (capped at a maximum intake contribution to 5-a-Day)	Soft drinks, not diet
Tea, coffee, and water	Soft drinks, diet
—	Alcoholic beverages
—	Sugar, preserves, and sweet spreads
—	Sugar confectionery
—	Chocolate confectionery

^1^Data categorized based on reference 30. NDNS RP, National Diet and Nutrition Survey Rolling Program.

We also selected a number of key foods and nutrients of public health interest in the UK population to examine in relation to eating location. Fruit and vegetables and fiber are consumed in insufficient amounts, whereas it is recommended that the intakes of red and processed meat, SSBs, nonmilk extrinsic sugars (NMESs) and SFAs should be reduced (32). Fruit and vegetable consumption values have been calculated using disaggregated data (33), and the number of portions have been calculated in line with the “5-a-Day” guidelines of 80 g/portion (including up to one 150-mL portion of fruit juice) for adults, which has been applied to children aged ≥5 y (34). For children aged ≤4 y this has been calculated as 40 g/portion (including up to one 75-mL portion of fruit juice) (35). SSBs include concentrated, still, and carbonated soft drinks with added sugar.

### Data management and statistical analysis

NDNS RP data are weighted to adjust for differences in sample selection and response. All of the analyses were carried out by using the survey package in R version 3.0.2 (36) in order to account for the stratification and clustering in the NDNS sample design.

Data for all children aged 1.5–18 y sampled between 2008 and 2014 were included. A descriptive analysis of the percentage of eating locations, core and noncore food consumption, and energy intake was carried out. Mean intakes in grams of fruit and vegetables, red and processed meat, SSBs, and fiber and as a percentage of total energy of NMESs and SFAs were calculated at each location. Nutrient (NMES and SFA) intakes are presented as the percentage of total energy intake to allow for comparisons to recommended daily energy intakes. Direct comparisons to current recommended intakes cannot be made by location for foods (fruit and vegetables, red and processed meats, and fiber) because these can only be presented as a proportion of overall food intake. Due to an unequal distribution of eating occasions per location, the intakes of selected foods and nutrients in each location, as a proportion of overall food/beverage (for foods) and energy (for nutrients), respectively, in that location, were calculated for each individual and are presented as population means. Comparisons between the intakes of selected foods and nutrients within each age group (1.5–3, 4–10, and 11–18 y) were carried out by using logistic regression, and patterns between age groups were compared for consistency. Given the public health interest with regard to the nutritional content of school meals compared with meals brought to school from home (15, 18), meal consumption at school was considered separately by food purchased at school compared with food brought from home. All recorded instances of food and beverages consumed at school were categorized as “school-sourced” or “home-sourced” whenever information about the source was available, regardless of time of eating. Eating occasions at schools for which this information was not available were excluded from this analysis (3.6% of food eaten at school). Comparisons of food and nutrients between school-sourced and home-sourced meals or foods within age groups were carried out by using linear regression, and consistency of patterns between age groups was compared.

To investigate the impact of modulating factors on eating location patterns and to gain an understanding of eating patterns, the sample was separated into quintiles by the percentage of consumption of meals at home as those consuming <25%, 25–49%, 50–69%, 70–89%, and ≥90% of meals at home. The home category was chosen after initial exploration of the data. Although home was the most frequent eating location, meals were also consumed in other locations; however, the small number of occasions did not allow for the characterization of these individual locations. We also considered that children who consumed ≥90% of meals at home are likely to differ from those who consume fewer meals at home (11). The intakes of SFAs, NMESs, fiber, and fruit and vegetables, which were chosen for their policy relevance as nutrition targets for the United Kingdom, were compared against recommendations by home meal-pattern category within each age group. We also examined the impact of potential confounders, such as age, sex, ethnicity, BMI, income, and drinking and smoking status (collected only in children aged ≥8 y) on home meal-pattern categories with the use of multiple linear regression analysis. Interactions between variables were analyzed when appropriate. The level of significance for all analysis was set at *P* < 0.05.

## RESULTS

**FIGURE 1:**
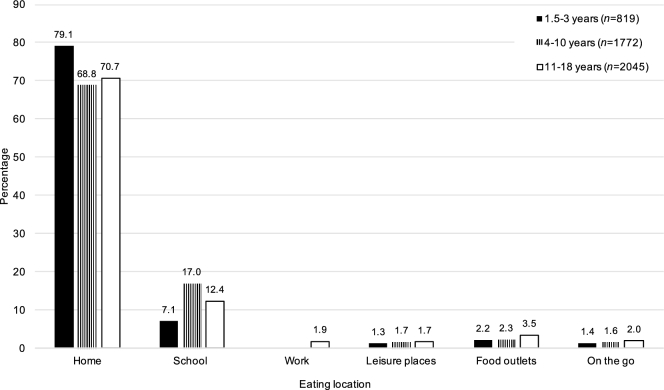
Percentage of reported eating location by age group for the NDNS RP years 1–6 (2008–2014) child population. NDNS RP, National Diet and Nutrition Survey Rolling Program.

The sample consisted of 4636 children (819 children aged 1.5–3 y, 1772 aged 4–10 y, and 2045 aged 11–18 y) with a total of 80,075 eating occasions. The main eating location across all age groups was home (68.8–79.1% of eating occasions) followed by school (7.1–17.0% of eating occasions) (**Figure 1**). The percentage of eating occasions in leisure places, food outlets, and “on the go” combined increased with age from 4.9% for children aged 1.5–3 y to 5.6% for children aged 4–10 y and 7.2% for children aged 11–18 y.

**FIGURE 2:**
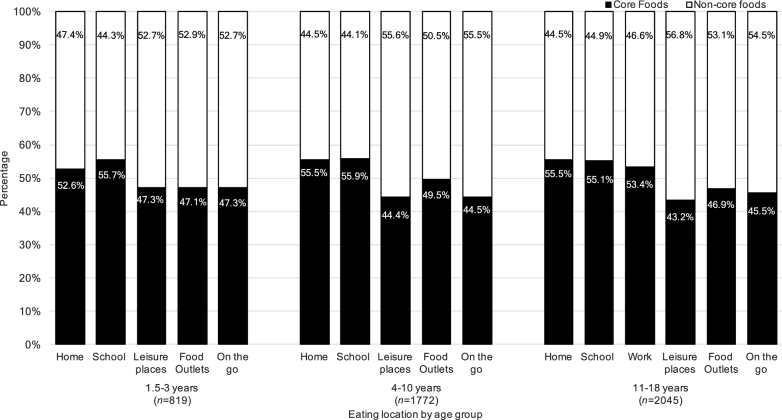
Percentage of consumption of core and noncore foods by reported eating location for the NDNS RP years 1–6 (2008–2014) child population. NDNS RP, National Diet and Nutrition Survey Rolling Program.

There was a higher percentage consumption of core foods at home and school in all age groups and at work for children aged 11–18 y than the other locations examined (food outlets, leisure places, and “on the go”), where a higher percentage consumption of noncore food was observed, especially in leisure places and “on the go” (**Figure 2**).

**FIGURE 3:**
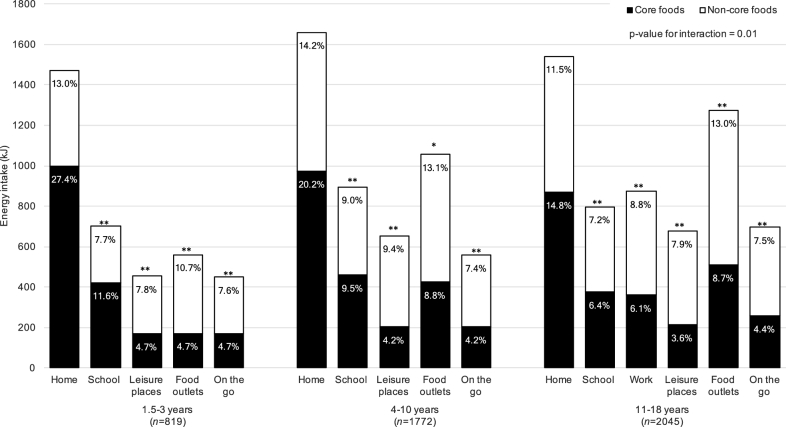
Energy intake from core and noncore foods by reported eating location for the NDNS RP years 1–6 (2008–2014) child population. Results of regression analyses comparing energy intake from core and noncore foods in all locations compared with at home are denoted as follows: **P < 0.01 and *P < 0.05. The consistency of energy intake from core and noncore foods across locations between age group was compared (presented as *P* values for interaction). Percentages represent energy intakes from both core and noncore foods by age group and add up to 100%. NDNS RP, National Diet and Nutrition Survey Rolling Program.

The 2 locations with the highest energy intake and highest noncore food energy intake were home and food outlets, with the exception of children aged 1.5–3 y for whom it was home and school. With regard to energy intake (**Figure 3**), the contribution of noncore foods increased with age, from 46.8% of mean energy intake in children aged 1.5–3 y up to 55.9% in children aged 11–18 y. Of all locations, home was the location with the highest contribution to core food energy intake, although this contribution decreased with age from 27.4% of energy intake in children aged 1.5–3 y to 14.8% of energy in children aged 11–18 y. Core food energy intake at school also decreased with age, from 11.6% in children aged 1.5–3 y to 6.4% in children aged 11–18 y. Although the overall mean core food energy intake was higher than noncore food energy intake in children aged 1.5–3 y, noncore foods contributed a higher percentage of energy than core foods in leisure places, food outlets, and “on the go”; the opposite was true for home and school in this age group. Similarly, for children aged 4–18 y, leisure places and food outlets combined contributed a high percentage of total daily energy intake (22.5%), with core foods only contributing 13.0%. Across all ages, eating “on the go” accounted for 4.2–4.7% of core food energy intake and close to double this from noncore foods (7.4–7.6%), which suggested a strong association between eating “on the go” and noncore food intake. Energy intake from core and noncore foods in all locations was significantly different from that at home, and energy intake across locations was significantly different across age groups (*P*-interaction = 0.01). Home was the only eating location where a higher percentage of energy was consistently consumed from core foods than from noncore foods in all age groups, whereas school contributed more or a similar proportion from core foods in children aged 1.5–10 y.

### Average intakes by eating location

Average intakes of selected foods and nutrients as a percentage of overall consumption by location are presented in **Table 2**.

**TABLE 2:** Consumption of selected foods and nutrients by reported eating location from the NDNS RP 2008–2014 as a percentage of overall intake by location and age group^1^

	Fruit and vegetables		Red and processed meat		Sugar-sweetened beverages		Fiber		NMESs		SFAs	
	Mean overall intake, %	*P*	Mean overall intake, %	*P*	Mean overall intake, %	*P*	Mean overall intake, %	*P*	Mean overall intake, %	*P*	Mean overall intake, %	*P*
Age 1.5–3 y (*n* = 819)												
Home	12.7	Ref	2.2	Ref	7.3	Ref	0.6	Ref	11.4	Ref	14.8	Ref
School	20.4	<0.001	1.3	<0.001	2.9	0.44	0.7	0.06	10.6	0.39	13.0	<0.001
Leisure places	12.8	0.98	4.2	0.15	6.4	0.29	0.8	0.01	21.4	<0.001	13.2	0.05
Food outlets	8.3	<0.001	3.3	0.03	14.3	<0.001	0.8	<0.001	18.4	<0.001	11.5	<0.001
On the go	17.6	<0.01	2.0	0.84	9.6	<0.001	0.7	<0.001	25.4	<0.001	12.3	<0.001
Age 4–10 y (*n* = 1772)												
Home	12.1	Ref	2.8	Ref	10.4	Ref	0.7	Ref	13.4	Ref	13.2	Ref
School	17.8	<0.001	2.7	0.34	6.8	0.04	0.8	<0.001	12.5	<0.01	13.0	0.39
Leisure places	4.9	<0.001	1.9	<0.01	22.4	<0.001	0.6	0.03	35.6	<0.001	10.1	<0.001
Food outlets	6.6	<0.001	3.6	0.04	24.5	<0.001	0.6	<0.01	20.0	<0.001	11.7	<0.001
On the go	12.4	0.76	1.7	<0.001	18.4	<0.001	0.9	<0.01	31.2	<0.001	12.2	0.02
Age 11–18 y (*n* = 2045)												
Home	10.0	Ref	3.4	Ref	17.3	Ref	0.7	Ref	13.7	Ref	12.6	Ref
School	9.4	0.13	3.0	0.04	20.2	<0.001	0.8	<0.001	16.5	<0.001	11.9	<0.01
Work	5.0	<0.001	2.9	0.40	18.5	<0.001	0.7	0.66	19.7	<0.01	10.7	<0.01
Leisure places	2.4	<0.001	1.2	<0.001	35.0	<0.001	0.5	<0.001	40.4	<0.001	8.1	<0.001
Food outlets	5.4	<0.001	3.6	0.24	31.0	0.03	0.6	<0.01	19.8	0.82	10.9	0.08
On the go	7.4	<0.01	2.3	<0.001	31.3	<0.001	0.8	<0.01	29.7	<0.001	10.8	<0.001
*P*-interaction	<0.001		<0.001		0.002		<0.001		<0.001		0.002	

^1^Results of linear regression are indicated under the respective *P*-value columns. The interaction between age group and selected foods and nutrients was tested by using the same models and is shown at the bottom of the table. NDNS RP, National Diet and Nutrition Survey Rolling Program; NMES, nonmilk extrinsic sugar; Ref, reference.

#### Fruit and vegetables

As a proportion of overall food intake, the highest fruit and vegetable consumption was at school followed by “on the go” in children aged 1.5–3 y, at school for children aged 4–10 y, and at home followed by at school for children aged 11–18 y. School was a frequent location of consumption in all age groups; nevertheless, fruit and vegetable consumption across all locations decreased with age (Figure 1, Table 2), and in older children (11–18 y) it was less than half that of the youngest group. Consumption in school was significantly higher than at home in children aged ≤10 y (*P* < 0.001). Consumption in leisure places (in children aged ≥4 y only) and in food outlets was significantly lower than at home (*P* < 0.001). The proportion of consumption by age group was significantly different across locations (*P* < 0.001).

#### Red and processed meat

The highest proportion was consumed in leisure places in children aged 1.5–3 y, whereas in children aged ≥4 y the highest proportion consumed was in food outlets. A similar proportion of red and processed meat was consumed at home and in school in children aged ≥4 y, whereas consumption in leisure places and “on the go” was significantly lower than at home in these age groups (*P* < 0.01). The proportion of consumption by age group was significantly different across locations (*P* < 0.001).

#### SSBs

The consumption of SSBs increased significantly with age across all locations. Leisure places and food outlets were the locations with the highest consumption across all age groups. Consumption in children aged 11–18 y was similarly high “on the go” and was higher at school and work than at home. Consumption in all locations was significantly higher than at home in children aged ≥4 y, with the exception of at school in children aged 4–10 y, where it was significantly lower (*P* < 0.001). The proportion of consumption by age group was significantly different across locations (*P* < 0.01).

#### Fiber

Fiber intake was low across all age groups, with only small differences by location. However, fiber intakes in this sample were low overall, with consumption remaining well below recommendations in all age groups (15–30 g/d depending on age group) (32). The proportion of consumption by age group was significantly different across locations (*P* < 0.001).

#### NMESs

NMES intake as a percentage of total energy increased across all locations with age and exceeded the recommended maximum of 11% of total energy (37). Home and school contributed the least to NMES intake, with higher intakes in leisure places, food outlets, and “on the go.” The contribution of NMES intake in leisure places, food outlets, and “on the go” was significantly higher than at home (*P* < 0.001), with the exception of food outlets in children aged 11–18 y. The proportion of consumption by age group was significantly different across locations (*P* < 0.001).

#### SFAs

Intake of SFAs as a percentage of total energy was highest in children aged 1.5–3 y and decreased with age across all locations. Intakes were comparable across locations, with only slightly higher intakes at home. The intakes exceeded the recommended maximum of 11% of total energy in all age groups and most locations (37). The proportion of consumption by age group was significantly different across locations (*P* < 0.01).

Overall, these results suggest that children consume red and processed meats, SSBs, and NMESs in excess when eating out of home, whereas SFAs are eaten in excess and fiber in insufficient amounts, irrespective of location. The consumption of fruit and vegetables was higher at school than other locations in school-aged children.

### School location

Eating at school was further categorized as occasions when food was purchased or obtained from the school (mostly from the school cafeteria) compared with when food was brought from home (mostly packed lunches) (**Table 3**). Analyses showed that energy intake tended to be lower from school-sourced food (“school meals”) than from home-sourced food (“packed lunches”) for children aged 4–10 y (mean ± SE, 1805 ± 40 compared with 1906 ± 38 kJ; *P* = 0.08). Protein intake was significantly higher in school-sourced meals in both age groups (*P* < 0.001), whereas NMES intake was lower in school-sourced meals, significantly so in children aged 4–10 y. Children aged 4–10 y had a lower intake of SFAs (*P* = 0.02) and a higher intake of fiber from school-sourced food (*P* < 0.001), whereas no significant differences were found between school- and home-sourced meals in children aged 11–18 y for intakes of these nutrients (saturated fats, *P* = 0.16; fiber, *P* = 0.80).

**TABLE 3:** Nutrient intake and food consumption from school meals and packed lunches consumed at school in children aged 4–18 y (attending full-time education) from the NDNS RP years 1–6 (2008–2014) child population^1^

	Age 4–10 y			Age 11–18 y			
	School- sourced meal	Home-sourced meal	*P*	School- sourced meal	Home-sourced meal	*P*	*P*-interaction
*n*	756	625		641	429		
Energy, kJ	1805 ± 40	1906 ± 38	0.08	2038 ± 55	1964 ± 65	0.38	0.09
Protein, % of total energy	15.5 ± 0.2	13.7 ± 0.2	<0.001	14.7 ± 0.3	12.6 ± 0.3	<0.001	0.56
Fat, % of total energy	32.8 ± 0.5	33.7 ± 0.6	0.19	33.3 ± 0.6	33.5 ± 1.0	0.85	0.55
SFAs, % of total energy	12.3 ± 0.3	13.3 ± 0.3	0.02	11.8 ± 0.3	11.2 ± 0.4	0.16	0.01
Carbohydrate, % of total energy	51.0 ± 0.6	51.8 ± 0.6	0.36	49.9 ± 0.8	52.8 ± 0.9	0.01	0.15
NMESs, % of total energy	10.7 ± 0.6	14.4 ± 0.6	<0.001	12.5 ± 0.7	14.9 ± 1.0	0.05	0.37
Fiber, g	3.7 ± 0.1	3.2 ± 0.1	<0.001	3.2 ± 0.1	3.2 ± 0.1	0.80	0.03
Fruit and vegetables, g	66.2 ± 2.5	59.3 ± 2.8	0.06	40.0 ± 2.6	48.2 ± 3.9	0.07	0.01
Red and processed meat, g	14.2 ± 1.0	14.4 ± 0.9	0.88	15.2 ± 1.3	13.2 ± 1.2	0.24	0.32
Sugar-sweetened beverages, g	195 ± 14	242 ± 13	<0.001	340 ± 15	310 ± 18	0.14	<0.001

^1^Values are means ± SEs unless otherwise indicated. Linear regression was used to compare between school-bought and home-bought meals within age groups. Consistency in patterns between age groups was also compared. NDNS RP, National Diet and Nutrition Survey Rolling Program; NMES, nonmilk extrinsic sugar.

Fruit and vegetable consumption in children aged 4–10 y tended to be higher from school-sourced meals than from home-sourced meals (*P* = 0.06), whereas in children aged 11–18 y, intakes tended to be slightly higher in home-sourced meals (*P* = 0.07). No differences were observed for red and processed meat consumption, with similar intakes from school- and home-sourced meals for children aged 4–10 y (*P* = 0.88) and 11–18 y (*P* = 0.24). Among participants who consumed SSBs, children aged 4–10 y who had home-sourced food had a higher consumption of SSBs than did those who had a school-sourced meal (*P* < 0.001), whereas for children aged 11–18 y, SSB consumption was similar across groups (*P* = 0.14). With regard to interactions, the differences in consumption between the 2 age groups by meal source were significant for intakes of SFAs, fiber, fruit and vegetables, and SSBs (*P* = 0.01, 0.03, and 0.01 and <0.001, respectively).

### Home eating consumption patterns and impact of modulatory factors

**FIGURE 4:**
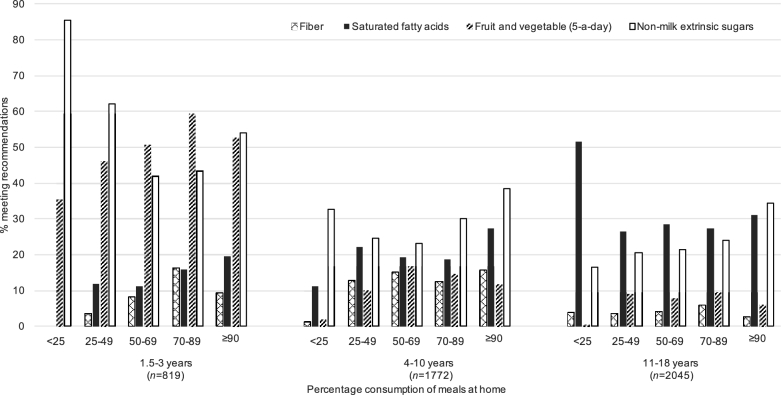
Percentage of NDNS RP years 1–6 (2008–2014) children meeting the recommended intakes of SFAs, nonmilk extrinsic sugars, and fiber according to home meal-consumption pattern. NDNS RP, National Diet and Nutrition Survey Rolling Program.

The comparison of intakes for SFAs, NMESs, fiber, and fruit and vegetables with recommendations by quintile of percentage of consumption of meals at home is presented in **Figure 4**. The percentage of children meeting the recommended intake of NMESs increased in those aged 11–18 y, with an increasing percentage of meals consumed at home. In younger children, the percentage meeting NMES intake followed a U-shaped pattern, with the lowest percentage meeting recommended intakes in those who consumed 50–69% of meals at home. There were small variations across the groups without a clearly defined pattern for other nutrients, with those consuming a higher percentage of meals at home being more likely to meet recommended intakes, especially fruit and vegetables in children aged 1.5–3 y.

Regression analyses of modulating factors showed that ethnicity (positive association for nonwhites), income, frequency of eating out, and frequency of takeaway meal consumption (negative association for higher income, eating out compared with not eating out, and eating takeaway meals compared with not eating takeaway meals) were significant factors affecting home consumption patterns (**Table 4**, model 1). Ethnicity (positive association) and frequency of eating out (negative association) remained significant predictors of a home-based meal consumption pattern when the model included drinking and smoking behavior in children aged ≥8 y for whom this information is collected (model 2). The effects of drinking and smoking frequency, however, were not significant. A higher proportion of children who consumed ≥90% of their meals at home were nonwhite (30.1%), whereas the split between white (82.6–91.9%) and nonwhite (8.1–17.4%) was comparable across the other percentage consumption groups. Similarly, the proportion of children increased across each percentage consumption group in those who reported never or rarely eating out (31.7%, 32.6%, 35.4%, 41.3%, and 46.8% across the percentage home consumption categories), whereas no clear pattern was seen across the other categories of eating out.

**TABLE 4:** Characteristics of the population by consumption pattern of meals at home for the NDNS RP years 1–6 (2008–2014) child population^1^

	Consumption of meals at home, %					*P*	
	<25	25–49	50–69	70–89	≥90	Model 1^2^	Model 2^3^
*n*	78	513	1556	1788	701		
Age, y	10.7	10.2	9.7	9.6	9.9		
Sex, %							
Male	40.7	48.4	50.8	53.4	49.7	Reference	Reference
Female	59.3	51.6	49.2	46.6	50.3	0.18	0.40
Ethnicity, %							
White	84.2	91.9	89.0	82.6	69.9	Reference	Reference
Nonwhite	15.8	8.1	11.0	17.4	30.1	<0.001	<0.001
Income (quintile), %							
≤£12,300	29.9	21.7	22.4	23.1	38.6	Reference	Reference
>£12,300 to ≤ £19,890	22.5	25.8	22.0	23.4	24.8	0.11	0.28
>£19,890 to ≤ £28,615	16.5	14.5	20.1	19.5	15.3	0.22	0.97
>£28,615 to ≤ £42,500	16.5	18.5	17.2	17.9	12.2	0.06	0.55
>£42,500	14.6	19.4	18.4	16.1	9.2	<0.01	0.21
BMI, %							
Normal weight	64.5	64.1	67.6	71.8	66.8	Reference	Reference
Overweight	16.6	17.8	14.7	12.4	16.0	0.14	0.58
Obese	18.9	18.1	17.8	15.8	17.2	0.27	0.08
Frequency of eating out, %							
≥5 times/wk	0.0	0.2	0.7	0.1	0.1	0.23	0.23
3–4 times/wk	1.8	0.7	0.5	1.1	1.8	<0.01	0.01
1–2 times/wk	17.3	22.8	19.6	19.4	17.5	<0.001	0.02
1–2 times/mo	49.3	43.7	43.8	38.1	41.3	<0.001	<0.01
Rarely or never	31.7	32.6	35.4	41.3	46.8	Reference	Reference
Frequency of takeaway meals, %							
≥5 times/wk	0.5	0.3	1.6	0.7	0.6	0.03	0.35
3–4 times/wk	1.6	2.0	1.8	1.3	0.9	0.20	0.28
1–2 times/wk	14.7	21.0	17.7	18.3	12.9	0.13	0.86
1–2 times/mo	55.5	51.7	55.0	49.7	37.2	<0.01	0.51
Rarely or never	27.8	25.0	23.9	29.9	48.4	Reference	Reference
Alcohol consumption,^4^ %							
Once a week or more	10.5	9.0	7.8	8.2	11.6		0.70
Once or twice a month	8.2	10.8	11.6	13.7	11.5		0.51
Once every couple of months	7.1	7.8	3.7	5.8	9.1		0.86
Few times a year	11.1	15.4	16.4	14.5	17.2		0.45
Never drinks	63.0	57.0	60.5	57.8	50.6		Reference
Smoking,^4^ %							
No	92	88.5	91.8	93.4	86.6		Reference
Yes	8.0	11.5	8.2	6.6	13.4		0.56

^1^Percentages within each category of percentage consumption of meals at home add up to 100% across subcategories for each variable and are interpreted by comparison across percentage consumption of meals at home groups. For example, for ethnicity, the split between white and nonwhite for the 4 percentage groups consuming <90% of meals at home was comparable (range: 82.6–91.9% for white and 8.1–17.4% for nonwhite), but for the >90% category, the split is of a different magnitude (69.9% white and 30.1% nonwhite) and implies that nonwhite children are more likely to eat more meals at home than are white children. NDNS RP, National Diet and Nutrition Survey Rolling Program.

^2^Model 1 includes sex, ethnicity, income, frequency of eating out, frequency of takeaway meal consumption, and BMI.

^3^Model 2 is for children aged ≥8 y because alcohol consumption and smoking information is collected only in this age group. The model includes sex, ethnicity, income, frequency of eating out, frequency of takeaway meal consumption, BMI, frequency of alcohol consumption, and smoking.

^4^Data collected for children aged ≥8 y.

In terms of proportions, girls were more likely to consume ≥90% of meals at home as were those from a nonwhite ethnic background (Table 4). Children in the lowest income quintile were most likely to consume ≥90% of their meals at home, although almost one-third of children in the same income quintile only consumed <25% of their meals at home. Children who consumed 50–69% of their meals at home were less likely to consume takeaway meals, with the likelihood decreasing with increasing percentage of consumption of meals at home. The category with the highest percentage of smokers was those who consumed ≥90% of their meals at home. More than 10% of children who consumed <25% or ≥90% of their meals at home reported consuming alcohol ≥1 times/wk.

## DISCUSSION

Our analysis of 4636 children involving >80,000 eating occasions shows that most of the energy intake in this nationally representative sample came from foods eaten at home. As children aged, they ate out of home and school (or work) more frequently and more energy came from less-healthy food options in these settings. Specifically, food outlets, leisure places, and “on the go” were the out-of-home food environments associated with the highest proportion of energy from noncore foods. For children aged 4–18 y, approximately one-third of total daily energy intake came from such foods in these locations, with core foods only contributing less than one-fifth in the same locations. A parallel analysis in adults aged ≥19 y from the NDNS RP showed a similar pattern, in that eating at food outlets, leisure places, and “on the go” was linked to higher energy intakes from noncore foods, with a disproportionately higher energy intake in these locations than at home and work (38). These results clearly highlight the potential impact that the immediate food environment can have on food choices in children and their potential effect in undermining health-promoting government messages.

The majority of eating occasions were at home across all age groups, and therefore this environment contributed the most to energy intake. Although the frequency of eating out increased with age, eating at home remained strongly related to healthier food intake. In line with previous findings, children in our analysis were more likely to have a higher core food energy intake than noncore food energy intake at home, suggesting that better dietary patterns are more likely when eating at home (8). In support of this, fruit and vegetable consumption at home in children has been associated with increased availability of these foods (39); however, parental supervision or rules about food and drink consumption may also play a role (40). The overall high percentage of eating occasions at home highlights the home as a potentially important target for public health policy through family and nutrition education or social marketing campaigns (e.g., Change4Life) and potentially the relevance of different strategies as children age and develop more independent eating habits (6). This is because food habits may be affected during adolescence with increased independence and social exposure; therefore, consideration of the wider, structural factors that shape such eating behaviors is also warranted. We found that among children aged ≤10 y, eating more meals at home was associated with meeting recommendations for SFAs, fruit and vegetables, and fiber, as well as for NMESs among adolescents, but this was not as clear for other nutrients. These findings suggest that public health campaigns recommending the consumption of more meals at home may need to be tailored to specific age groups.

After eating at home, school was the location with the highest percentage of eating occasions. Energy intake from core foods was higher than from noncore foods across all ages but the percentage of energy from noncore foods at school increased with age, as seen across all locations. Thus, as children aged, the proportion of energy from core and noncore foods in school tended to equalize. This was further confirmed in our analysis by food source at school, which showed that eating school-sourced foods, compared with home-sourced foods, was linked to overall better energy and nutrient content in children aged 4–10 y, whereas for children aged 11–18 y, intakes of fruit and vegetables, red and processed meat, and SSBs were similar for both types of meals. Our findings suggest that the school food environment is more protective for younger children than for older children, probably as a result of the uptake of government policies such as free school meals for low-income children and free fruit and vegetable snacks for all children aged 4–6 y. As children age, their independence and freedom of choice increase, which is facilitated by the school structure including more flexible school meal services (41, 42) and the freedom to purchase and choose foods outside the school (18, 43). The lower intake of fruit and vegetables among children aged 11–18 y may also reflect the fact that they are not included in the school 5-a-Day scheme and therefore these children are less exposed to fruit and vegetables. Overall, these results highlight the importance of continuing to support school initiatives around healthy food (44) and the challenge of encouraging smart choices through development and adolescence.

With regard to foods eaten in locations outside of the home and school, our study confirms previous findings (8, 11), which reflect the general lack of access to affordable nutrient-rich food in these locations. For instance, fruit and vegetable consumption decreased with age in leisure places and food outlets. Intakes of NMESs and SFAs consumed “on the go” in leisure places and food outlets were high, especially in older children, which may reflect increased mobility and independence of food choices (43). SSBs (a high contributor to NMES intake) were mainly consumed in leisure places and food outlets. The consumption of SSBs in these settings, together with fast food, is associated with net increases in daily energy intake (of ≤310 kcal in children aged 12–19 y) (4).

The specific mechanisms by which eating location influences food choice are not fully understood and are likely to include a wide range of factors, including behavioral, social, and environmental. The particular eating location itself may also favor the clustering of specific food behaviors. This may be due to the range of foods offered, which may be limited due to perishability, closeness to school or work, taste preferences, and cost of food. For example, children consume higher-fat foods when away from home (45) or school, which may be linked to higher salt content, causing low satiation but increased thirst and higher consumption of SSBs (43). In addition, families who consume meals out of home are also more likely to eat takeaway food at home (11), which may reflect a lack of time or skill for cooking or difficulty in synchronizing schedules of family members (46). In those who consume ≤25% of their meals at home, close to one-third belonged to the least-affluent families, which could be an indicator of either extreme poverty (eating regularly only when at school) (47) or commonly resorting to very cheap, nutrient-poor food. Although previous research has suggested a positive association between exposure to takeaway food outlets and BMI in adults (20), a similar association was not evident from our analysis. In support of this, a recent study in children suggested that BMI was also not associated with fast-food consumption after adjustment for age and sex; however, those who consume fast food >4 times/wk preferred larger portions of chips, which could lead to increased weight gain (43).

The present analysis used a nationally representative sample of children in the United Kingdom and explored a wide range of eating locations as well as food and nutrient sources, providing evidence for a link between eating location and consumption patterns. We also provide evidence of a switch from healthier food to less-healthy food consumption along the way to adulthood and independence. This can also serve as baseline data for future public health policy impact analyses, such as on school meal quality (48).

Our study has some limitations, including that the core and noncore food classification may not be specific enough—for example, the inclusion of fruit juice, sweetened dairy products, and refined grains as core foods (32). However, sweetened dairy products represented a very small proportion of all dairy products consumed and the impact of their inclusion within core foods is likely to be small. Coding for fruit juice intake was capped at the recommended portion of 150 mL/d in accordance with the UK government “5-a-Day” guideline. Although refined grains contribute less fiber and nutrients than do whole grains and tend to dominate dietary intakes, their inclusion as a core food reflects a consensus that they are important in the diet of children due to improved taste and texture, acting as vehicles for the intake of other nutrients such as protein and unsaturated fats (49). Another limitation was the inability to control for physical activity, peer or social pressure influences, dieting practices, or illness and exposure to food advertising, which could affect food choices and intakes. Although data on whether the television was on or off while eating were collected, information on what was being watched was not recorded. The impact of different types of food services on school lunches could also not be explored due to a lack of data. These and other variables representing the community food environment surrounding schools, homes, and the variety of locations where people consume food could not be cross-analyzed in the present study because this information is not available in order to maintain anonymity. Purchase location was not a primary outcome, and thus the study was not designed to optimally measure food purchases. Future research should explore these alongside potential interactions between foods or nutrients and specific settings. The effects of additional variables related to socioeconomic status, such as parental education, were not included, but this information was only available in <30% of participants. Finally, as with all self-reported dietary data, a degree of reactivity to food recordkeeping (50) and other dietary misreporting cannot be excluded, especially for the older children.

In conclusion, although energy intake from core foods was higher at home across all age groups, eating out-of-home, particularly in food outlets, leisure places, and “on the go,” was linked to higher energy intakes from noncore foods. The contribution of noncore foods to energy intake increased with age at the detriment of core food intake, which is potentially associated with increased independence, eating outside the home or school, and higher vulnerability to external food cues. Our results confirm that access to healthy food as part of school initiatives is an important factor to improve dietary choices in young children, with the secondary school environment warranting particular attention. At the same time, the lack of affordable healthy options in leisure places and outlets selling food to eat “on the go” may also act as a barrier to healthy eating, especially in older children. Although the high percentage of eating occasions at home highlights a potentially important target for intervention through family eating behaviors, our study further highlights a need to focus on improving food choices for older children in food environments outside of the home and school.

## Notes

The National Diet and Nutrition Survey Rolling Program is jointly funded by Public Health England and the UK Food Standards Agency. NZ, SN, PP, and EA-R were supported by the UK Medical Research Council (program U1059600384). EA-R acknowledges support from the Center for Nutrition Research, University of Navarra; the Spanish Biomedical Research Centre in Physiopathology of Obesity and Nutrition (CIBERobn), Institute of Health Carlos III, Madrid, Spain; and the Navarra Institute for Health Research (IdiSNa), Pamplona, Spain. SFLK acknowledges support from a Canadian Institutes of Health Research (CIHR) Canada Research Chair (2007–2016). TLP's PhD studentship was supported by the Cambridge International Scholarship, a scheme funded by the Cambridge Commonwealth, European and International Trust.

Abbreviations used:

CAPI: Computer-Assisted Personal Interview
DLW: doubly labeled water
NDNS: National Diet and Nutrition Survey
NMES: nonmilk extrinsic sugar
RP: Rolling Program
SSB: sugar-sweetened beverage
